# DIALIGN P: Fast pair-wise and multiple sequence alignment using parallel processors

**DOI:** 10.1186/1471-2105-5-128

**Published:** 2004-09-09

**Authors:** Martin Schmollinger, Kay Nieselt, Michael Kaufmann, Burkhard Morgenstern

**Affiliations:** 1 Wilhelm-Schickard-Institut fur Informatik, Sand 14, 72076 Tübingen, Germany; 2 Center for Bioinformatics Tübingen, Sand 14, 72076 Tübingen, Germany; 3 University of Göttingen, Institute of Microbiology and Genetics, Goldschmidtstr. 1, 37077 Göttingen, Germany

## Abstract

**Background:**

Parallel computing is frequently used to speed up computationally expensive tasks in Bioinformatics.

**Results:**

Herein, a parallel version of the multi-alignment program DIALIGN is introduced. We propose two ways of dividing the program into independent sub-routines that can be run on different processors: (*a*) pair-wise sequence alignments that are used as a first step to multiple alignment account for most of the CPU time in DIALIGN. Since alignments of different sequence pairs are completely independent of each other, they can be distributed to multiple processors without any effect on the resulting output alignments. (*b*) For alignments of large genomic sequences, we use a heuristics by splitting up sequences into sub-sequences based on a previously introduced *anchored alignment *procedure. For our test sequences, this combined approach reduces the program running time of DIALIGN by up to 97%.

**Conclusions:**

By distributing sub-routines to multiple processors, the running time of DIALIGN can be crucially improved. With these improvements, it is possible to apply the program in large-scale genomics and proteomics projects that were previously beyond its scope.

## Background

Multiple sequence alignment continues to be an active field of research in Computational Biology and a number of novel approaches have been developed during the last years, see [1] for an overview on multi-alignment algorithms and [2,3] for systematic evaluation of the commonly used software tools. Until some years ago, research on sequence alignment was mainly concerned with aligning proteins or single genes. During the last few years, however, comparison of genomic sequences became a crucial tool for uncovering functional elements such as genes or regulatory sites. Consequently, the focus of alignment research shifted to large genomic sequences [4,5]. Alignment of sequences in the order of hundreds of kilobases or megabases is computationally demanding. Some extremely efficient tools have been developed that are able to align entire chromosomes or genomes [6,7]. These approaches, however, work best on closely related species; they are unable to compare sequences with larger evolutionary distances.

DIALIGN [8] is a versatile tool for pair-wise and multiple alignment of nucleic acid and protein sequences. It combines global and local alignment features and is therefore particularly useful to align distantly related sequences sets sharing isolated local homologies. In a number of recent research projects DIALIGN has been used to align syntenic genomic sequences; some new program options have been implemented for this purpose [9]. Recent applications of DIALIGN in comparative genomics include detection of regulatory elements by multiple alignment [10-14], phylogenetic studies [15,16] and identification of signature sequences to detect pathogenic viruses as part of the US biodefense program [17]. An independent study by Pollard *et al*. evaluated the capability of alignment programs to detect conserved non-coding sites in genomic sequences. These authors conclude that *DIALIGN can produce alignments with high coverage and sensitivity, as well as specificity to detect constrained sites *[3].

Though DIALIGN produces alignments of high quality, it is slower than alternative multi-alignment programs. Especially if large genomic sequences are to be aligned DIALIGN is far more time-consuming than the above mentioned specialized programs for genomic alignment. A recently introduced *anchored alignment *option [18] can be used to speed-up the program, but even with this improvement DIALIGN is still slower than alternative software tools. Parallel computing has been used by various researchers in order to improve the running time of computationally expensive alignment procedures, see for example [19-21]. Herein, we introduce a parallel version of DIALIGN. We apply two different strategies to distribute sub-routines to multiple processors. In our test examples, the running time of DIALIGN could be reduced by up to 94.5 % for multiple protein alignment and by up to 97.5 % for alignment of large genomic sequences.

## Implementation

### Parallel multiple alignment

For multiple alignment, the DIALIGN algorithm works as follows: in a first step, all respective optimal pair-wise alignments are carried out. This means that, for each pair of input sequences, a *chain *of local *fragment alignments *with maximum total *weight score *is identified. A *fragment *or *fragment alignment *is defined as an un-gapped local pair-wise alignment, and the *weight score *of such a fragment is calculated based on a P-value i.e. on the probability of its random occurrence, see [8] for a detailed explanation of this approach. The chaining algorithm that identifies a fragment chain with maximum total weigth is described in [22].

For a set of *N *input sequences, *N *× (*N *- 1)/2 pair-wise alignments are to be calculated; fragments contained in these pair-wise alignments are then used to build up a multiple alignment in greedy fashion. If the maximum sequence length is bounded by some constant, the time-complexity of this algorithm as a function of the number *N *of sequences is as follows: Performing all pair-wise alignments takes *O*(*N*^2^) time. During the greedy procedure, *O*(*N*) independent fragments can be included into the multiple alignment; additional fragments would be either inconsistent or already contained in the existing multiple alignment. Accepting a single fragment takes *O*(*N*^2^) time since for each accepted fragment, so-called *consistency *frontiers are to be updated. These frontiers are used to decide if subsequent fragments are consistent with previously accepted fragments. Thus, the worst-case time complexity of our multi-alignment algorithm is *O*(*N*^3^). Test runs with real data show, however, that the real time-complexity is something between quadratic and cubic, see [23] for a detailed analysis of the complexity and running time of our algorithm.

The current version of DIALIGN uses an efficient algorithm to update the consistency frontiers. This means that, although performing all pair-wise alignments has a lower theoretical time complexity than processing the fragments from these alignments in the greedy algorithm, for realistic data sets most of the CPU time is spent on the pair-wise alignments. For example, for a set of 20 protein sequences with an average length of 367 amino acid residues, as much as 97.4 % of the CPU time is used to perform the 20 × 19/2 = 190 pair-wise alignments. However, the *relative *proportion of CPU time used for pair-wise alignments *decreases *with the number of input sequences, as can be expected from the above theoretical considerations.

The pair-wise alignments that are calculated as the first step of the multi-alignment procedure are completely independent of each other. Thus, it is obvious that the total program running time can be crucially reduced by running these procedures on parallel processors. Here, an important point is to distribute the work load evenly to the different processors in order to minimize the total program running time. To this end, our algorithm first estimates the running time for each pair-wise alignment as a function of the sequence length. As outlined in [22], the running time of DIALIGN for pair-wise alignment is proportional to the product of the sequence lengths. Based on this estimate, the algorithm distributes the *N *× (*N *- 1)/2 pair-wise alignments to the available processors in order to balance the work load. Here, we are using a greedy algorithm to find a satisfactory work-load distribution in reasonable time: we first assign a processor to the pair-wise alignment with the longest expected running time, then assign a processor to the second-largest pairwise alignment etc.

### Alignment of large genomic sequences

The quadratic time complexity of the original DI ALIGN algorithm for pair-wise alignment is clearly not efficient enough to align large genomic sequences. To improve the running time of DIALIGN for long sequences, an *anchored alignment *procedure has recently been implemented [18,24]. For our parallel approach, we use the fast local alignment tool *CHAOS *to identify a *chain *of high-scoring local alignments for each pair of input sequences . To select a *consistent *sub-set of these local alignments, a greedy algorithm is used, see [18] for details.

CHAOS uses a *trie *data structure to identify pairs of segments with a user-defined upper bound on the number of mismatches per segment pair. It defines a local alignment as a *chain *of such gap-free local alignments that are located within a certain distance from each other. Finally, the algorithm returns an optimal chain of such local alignments. After the consistency check, these alignments are used by our algorithm to define *anchor points *in order to narrow down the search space for the final pair-wise alignment procedure that is performed by DIALIGN as explained in [18].

**Figure 1:**
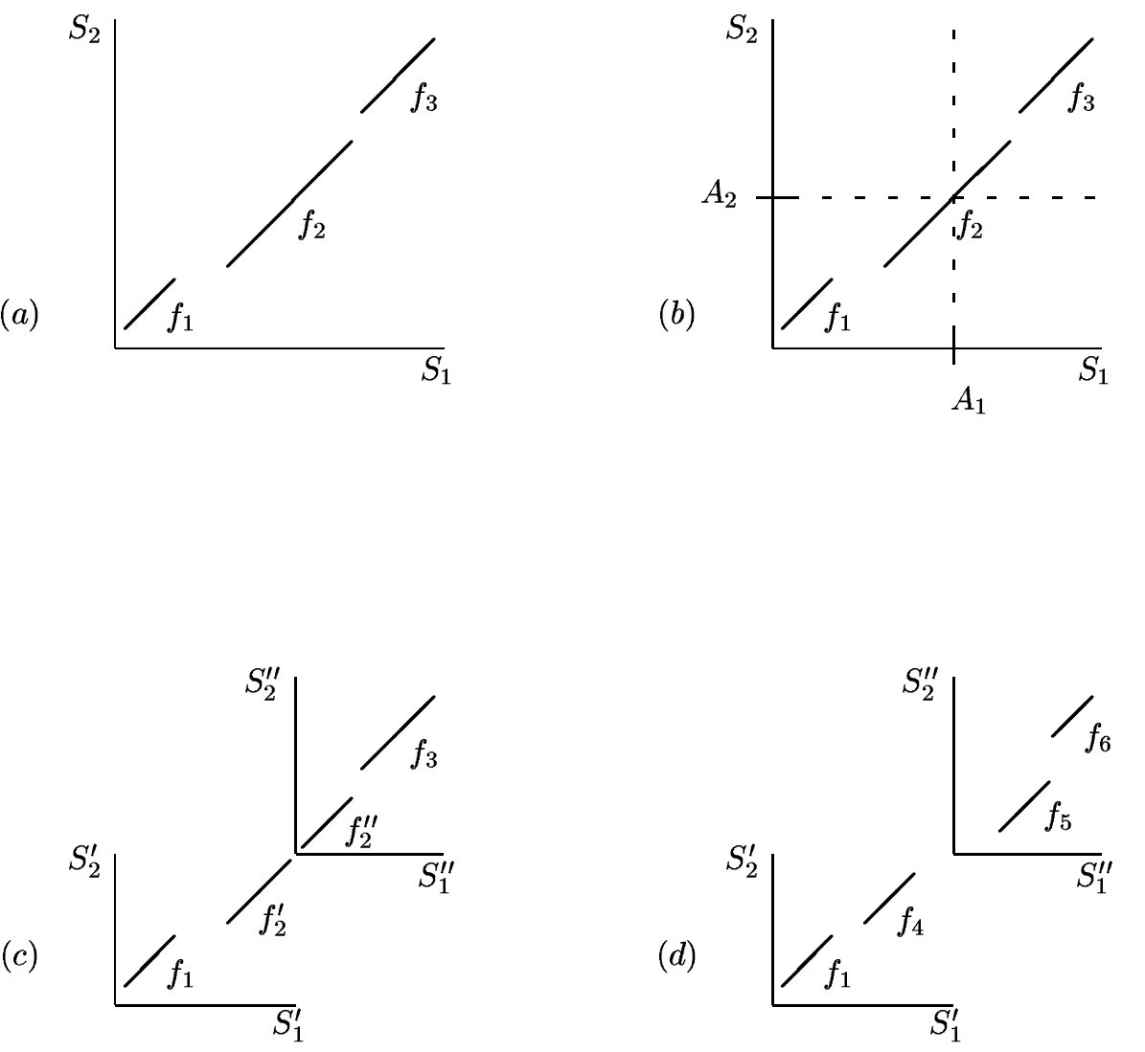
**Anchored alignment and parallel computation of sub-alignments for pair-wise sequence comparison. **(*a*) Un-constrained alignment: an optimal chain of fragments *f*_1_, *f*_2_, *f*_3 _is identified in the comparison matrix spanned by input sequences *S*_1 _and *S*_2_. (*b*) Anchored alignment: an anchor point (*A*_1_, *A*_2_) is defined prior to the alignment procedure, and an optimal chain of fragments is searched under the constraints imposed by this anchor. That is, residues to the left of *A*_1 _can be aligned only to residues to the left of *A*_2 _and vice versa, so the alignment search space is reduced by a factor of 2. If anchor point (*A*_1_, *A*_2_) is *consistent *with the optimal un-constrained alignment as shown in (*a*), the constraints imposed by this anchor point do not affect the resulting alignment, so exactly the same fragment chain *f*_1_, *f*_2_, *f*_3 _is obtained. (*c*) In the *parallel *aligment procedure, sequence *S*_1 _and *S*_2 _are split into sub-sequences 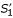, 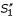 and 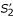, 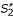, respectively, where the anchor point (*A*_1_, *A*_2_) defines the exact cutting-position. Alignments of 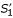 and 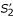 and of 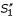 and 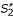 are calculated independently, and concatenated to obtain an output alignment of *S*_1 _and *S*_2_. In (*c*), the obtimal sub-alignments involve fragments 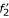 and 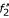 which are sub-fragments of *f*_2 _contained in the un-constrained alignment. In this case, the resulting final alignment of sequences *S*_1 _and *S*_2 _is therefore the same as the un-constrained one in example (*a*). Note, however, that the scoring function *w *for fragments is *not additive *and we may have, for example, 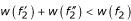. Therefore, even if anchor point (*A*_1_, *A*_2_) is consistent with the optimal un-constrained alignment, there is no guarantee that concatenating optimal sub-alignments yields the same alignment as the un-constrained alignment of the input sequences *S*_1 _and *S*_2_. The algorithm may end up with a situation as shown in example (*d*) where the parallel alignment procedure leads to a different output alignment.

To be precise, an anchor point is a pair of segments, one segment from each of the two input sequences; this way each position in the first segment is assigned to the respective position in the second segment. If a residue *x *is assigned to a residue *y *through one of the anchor points, this means that *x *is the *only *residue that can be aligned with *y *in the final output alignment. Whether or not *x *and *y *will be aligned depends on the degree of local sequence similarity that DIALIGN detects. Moreover, all residues to the left of *x *can be aligned only with residues to the left of *y *and vice versa, see Figure 1. The algorithm then returns an optimal alignment, i.e. a chain of fragments with maximum total weight score respecting the constraints imposed by the selected anchor points. Note that, if all anchor points are consistent with the optimal *non-anchored *alignment, then the result of the anchored alignment procedure will necessarily be the same as for the non-anchored procedure. In particular, this is the case if all anchor points are *part *of the optimal non-anchored alignment.

In the present study, we use selected anchor points as *cutting positions *to split the input sequences into smaller *sub-sequences*, and we reduce the program running time for DIALIGN by aligning these sub-sequences *independently *on multiple processors. This procedure is related to Stoye's well-known *divide-and-conquer *approach to multiple alignment [25] and to the linear-space algorithm for pair-wise alignment proposed by Hirschberg [26].

It should be mentioned that, unlike the above outlined anchoring procedure, distributing sub-alignments to multiple processors may well affect the resulting output alignments, even if the selected cut positions are consistent with the optimal alignment. No matter how well our anchoring positions are chosen, we can generally not expect the optimal alignments of our sub-sequences to coincide with the optimal alignment of the original input sequences The reason for this behaviour is that DIALIGN uses a *non-additive *weighting function *w *for segment pairs (fragments). If a large fragment *f *is split into two smaller sub-fragments *f*_1_, *f*_2_, the sum *w*(*f*_1_) + *w*(*f*_2_) is, in general, lower than the original weight *w*(*f*), see [8]. As demonstrated in Figure 1, concatenating alignments of sub-sequences may or may not result in the optimal alignment that would be returned by the naive alignment procedure or by the anchored procedure if anchor points are selected appropriately. Thus, special care has to be taken in selecting appropriate sub-sequences. In particular, the number of splits should not be too high as every split can possibly reduce the quality of the output alignment.

To reduce the total running time of the alignment procedure as far as possible, our algorithm distributes the sub-sequence alignments evenly to the available processors. At the same time, it minimizes the loss of alignment quality that may be caused by splitting the sequences into too many subsequences. To this end, we first identify a chain of anchor points using CHAOS. The program then divides the sequences at every anchor point, thereby producing a large set S1 of pairs of relatively small sub-sequences. The running time for each of the corresponding sub-alignments is estimated as described above. Using these estimates, the sub-sequences in S1 are concatenated in such a way that a set S2 of larger sub-sequences is obtained that can still be evenly distributed to the available processors. Here again, we use a greedy approach to assign processors to sub-alignments. As a result, our algorithm minimizes the program running time by balancing the work load among the processors while it maximizes the length of the aligned sub-sequences, thereby reducing the possible loss of alignment quality.

### Computer resources

We decided to use the message-passing interface (MPI) [27,28] for our work. Efficient MPI libraries are available for all supercomputing systems, and also for casual workstation pools. The results reported in the next section were achieved by experimental tests made on the Kepler-Cluster [University of Tübingen (SFB-382, ) that is a Linux-SMP cluster with two Pentium III processors (650 MHz) and 1 GB main memory per node. The nodes are connected by a Myrinet 1.28 GBit/s switched LAN. The software was also compiled and tested on a Sun fire with 8 processors and on an ordinary Linux-based workstation pool that generally exists in every institute.

## Results and conclusion

The performance of existing multi-alignment software has been evaluated in detail. All programs have been extensively tested by their authors; in addition several independent studies have been carried out using numerous sets of real and artificial benchmark data. The quality of multiple-protein-alignment programs including DIALIGN has been systematically studied by Thompson *at al*. [29] and by Lassmann and Sonnhammer [2]. The ability of multi-alignment tools to detect conserved patterns in *genomic *sequences has recently been investigated by Pollard *et al*. [3]. Since the goal of the present study is to speed-up an *existing *approach, we do not evaluate the *quality *of the produced output alignments; the ability of our software to produce biologically meaningful alignments under various conditions has been evaluated in the above cited papers. Herein, we compare the *running time *of our parallel software to the original serial version of the program.

As a first test example, we aligned sets of proteins with 20, 55, and 100 sequences, respectively. The program distributed the pair-wise alignments to different processors as described in section. Table 1 shows running time and speed-up for different numbers *p *of processors. Using 64 processors, the running time for these data sets can be reduced by 94.82%, 90.80% and 75.90%, respectively, compared to the serial running time. The observed differences in the relative speed-up are due to the different proportion of CPU time that is spent on the pairwise alignments, see [23]. The larger this proportion is, the higher is the relative speed-up that can be achieved by running these procedures on parallel processors (Amdahl's law [30]). Further improvements in program running time should be possible by parallelizing other parts of the algorithm such as fragment sorting and consistency calculations during assembly of the multiple alignment [23].

Next, we looked at the improvement of program running time that can be achieved for large genomic sequences using the algorithm described in section. As a test example, we used a set of three syntenic genomic sequences from mouse, rat and human. Each of these sequences is around 1 MB in length. The program CHAOS identified a total of 15,818 anchor points; 4,294 for human/mouse, 4,072 for human/rat and 7,452 for mouse/rat. Most of these anchor points were consistent with each other, only 121 out of the 15,818 anchor points had to be discarded because of consistency problems [18]. The consistent anchor points led to a set S1 containing 15,700 pairs of sub-sequences with an average length of 214 *bp *(note that, for each sequence pair, a chain of *n *anchor points divides the sequences into *n *+ 1 pairs of sub-sequences). These pairs of subsequences were concatenated to obtain a set S2 of larger sub-sequences that can be evenly distributed to the available processors.

Using CHAOS anchor points, the serial version of our program took 267,574 s = 74 h 19 m 34 s to compute the multiple alignment of our input sequences on a single processor of our cluster. We estimate that, without the anchoring option, the original DIALIGN program would have taken around three weeks to align these sequences. By contrast, the parallel version of the program with 64 processors took only 6,583 s = 1 h 49 m 43 s to align the same sequence set, corresponding to a running time improvement by 97.5 %. With a speed-up from more than three days to less than two hours, DIALIGN can now compute long-range multiple alignments of genomic sequences that were, until recently, far beyond its scope.

## Availability

The program will be available online through *Göttingen Bioinformatics Compute Server (GO-BICS) *at . The program code is available on request.

## Authors' contributions

MS parallelized DIALIGN, installed it on the Kepler cluster, performed the test runs and wrote parts of the manuscript. KN and MK participated in the design of the project and manuscript preparation. BM supervised the project and wrote most of the manuscript. All authors read and approved the final manuscript.

## Figures and Tables

**Table 1:** Test results for three data sets using different numbers of processors *p *Running time of the parallelized version of DIALIGN on protein sequences (average length: 381 residues) using different numbers *p *of processors. The values for *p *= 1 are measured with DIALIGN, the others with ParDIALIGN. All running times in the table are the fastest achieved values of several runs. The speed-up value is defined as the time needed for *p *= 1 divided by the time for ParDIALIGN. The percentages show the reduction of the time with respect to the sequential version of DIALIGN.

	20 sequences		55 sequences		100 sequences	
*p*	time (sec)	speed-up	time (sec)	speed-up	time (sec)	speed-up

1	37.69	-	334.17	-	1283.80	-
4	10.40	3.62 (72.41%)	104.61	3.19 (68.70%)	552.68	2.32 (56.95%)
8	5.93	6.36 (84.27%)	65.69	5.09 (80.34%)	421.69	3.04 (67.15%)
16	3.57	10.56 (90.53%)	46.25	7.23 (86.16%)	359.50	3.57 (72.00%)
32	2.65	14.22 (92.97%)	36.92	9.05 (88.95%)	332.22	3.86 (74.12%)
64	1.95	19.32 (94.82%)	30.74	10.87 (90.80%)	309.43	4.15 (75.90%)

## References

[B1] Notredame C (2002). Recent progress in multiple sequence alignment: a survey. Pharmacogenomics.

[B2] Lassmann T, Sonnhammer EL (2002). Quality assessment of multiple alignment programs. FEBS Letters.

[B3] Pollard DA, Bergman CM, Stoye J, Celniker SE, Eisen MB (2004). Benchmarking tools for the alignment of functional noncoding DNA. BMC Bioinformatics.

[B4] Chain P, Kurtz S, Ohlebusch E, Slezak T (2003). An applications-focused review of comparative genomics tools: capabilities, limitations, and future challenges. Briefings in Bioinformatics.

[B5] Miller W (2001). Comparison of genomic DNA sequences: solved and unsolved problems. Bioinformatics.

[B6] Salzberg SL, Delcher AL, Kasif S, White O (1998). Microbial gene identification using interpolated Markov models. Nucleic Acids Research.

[B7] Hohl M, Kurtz S, Ohlebusch E (2002). Efficient multiple genome alignment. Bioinformatics.

[B8] Morgenstern B (1999). DIALIGN 2: improvement of the segment-to-segment approach to multiple sequence alignment. Bioinformatics.

[B9] Morgenstern B, Rinner O, Abdeddaïm S, Haase D, Mayer K, Dress A, Mewes HW (2002). Exon Discovery by Genomic Sequence Alignment. Bioinformatics.

[B10] Göttgens B, Barton L, Gilbert J, Bench A, Sanchez M, Bahn S, Mistry S, Grafham D, McMurray A, Vaudin M, Amaya E, Bentley D, Green A (2000). Analysis of vertebrate SCL loci identifies conserved enhancers. Nature Biotechnology.

[B11] Göttgens B, Gilbert J, Barton L, Grafham D, Rogers J, Bentley D, Green A (2001). Long-range comparison of human and mouse SCL loci: localized regions of sensitivity to restriction endonucleases correspond precisely with peaks of conserved noncoding sequences. Genome Res.

[B12] Göttgens B, Barton L, Chapman M, Sinclair A, Knudsen B, Grafham D, Gilbert J, Rogers J, Bentley D, Green A (2002). Transcriptional regulation of the Stem Cell Leukemia gene (SCL) Comparative analysis of five vertebrate SCL loci. Genome Res.

[B13] Guo H, Moose SP (2003). Conserved noncoding sequences among cultivated cereal genomes identify candidate regulatory sequence elements and patterns of promoter evolution. Plant Cell.

[B14] Chapman MA, Charchar FJ, Kinston S, Bird CP, Grafham D, Rogers J, Grützner F, Graves JAM, Green AR, Göttgens B (2003). Comparative and functional analysis of LYL1 loci establish marsupial sequences as a model for phylogenetic footprinting. Genomics.

[B15] Prohaska S, Fried C, Flamm C, Wagner GP, Stadler PF (2004). Surveying Phylogenetic Footprints in Large Gene Clusters: Applications to Hox Cluster Duplications. Mol Evol Phylog.

[B16] Fried C, Prohaska S, Stadler P (2003). Independent Hox-cluster duplications in lampreys. J EXP ZOOL PART B.

[B17] Fitch J, Gardner S, Kuczmarski T, Kurtz S, Myers R, Ott L, Slezak T, Vitalis E, Zemla A, McCready P (2002). Rapid Development of Nucleic Acid Diagnostics. Proceedings of the IEEE.

[B18] Brudno M, Chapman M, Göttgens B, Batzoglou S, Morgenstern B (2003). Fast and sensitive multiple alignment of large genomic sequences. BMC Bioinformatics.

[B19] Yap TK, Frieder O, Martino RL (1998). Parallel Computation in biological sequence analysis. IEEE Transactions on Parallel and Distributed Systems.

[B20] Kleinjung J, Douglas N, Heringa J (2002). Parallelized multiple alignment. Bioinformatics.

[B21] Li KB (2003). ClustalW-MPI: ClustalW analysis using distributed and parallel computing. Bioinformatics.

[B22] Morgenstern B (2002). A simple and space-efficient fragment-chaining algorithm for alignment of DNA and protein sequences. Applied Mathematics Letters.

[B23] Abdeddaïm S, Morgenstern B (2001). Speeding up the DIALIGN multiple alignment program by using the 'Greedy Alignment of BIOlogical Sequences LIBrary' (GABIOS-LIB). Lecture Notes in Computer Science.

[B24] Morgenstern B, Prohaska SJ, Werner N, Weyer-Menkhoff J, Schneider I, Subramanian AR, Stadler PF Multiple sequence alignment with user-defined constraints. In Proceedings GCB'04, Lecture Notes in Informatics.

[B25] Stoye J (1998). Multiple sequence alignment with the divide-and-conquer method. Gene.

[B26] Hirschberg DS (1975). A linear space algorithm for computing maximal common subsequences. Commun ACM.

[B27] Message Passing Interface Forum (1994). MPI: A Message-Passing Interface Standard. Tech Rep CS-94-230, Computer Science Department, University of Tennessee, Knoxville, TN.

[B28] Message Passing Interface Forum (1997). MPI-2: Extensions to the Message-Passing Interface. Tech rep, Computer Science Department, University of Tennessee, Knoxville, TN.

[B29] Thompson JD, Plewniak F, Poch O (1999). A comprehensive comparison of protein sequence alignment programs. Nucleic Acids Research.

[B30] Amdahl GM (1967). Validity of the single processor approach to achieve large-scale computing capabilities. In AFIPS Conference Proceedings 30.

